# Continuous renal replacement therapy rescued life-threatening capillary leak syndrome in an extremely-low-birth-weight premature: a case report

**DOI:** 10.1186/s13052-021-01067-8

**Published:** 2021-05-26

**Authors:** Li-Fen Yang, Jia-Chang Ding, Ling-Ping Zhu, Li-Xia Li, Meng-Qi Duan, Zhuang-gui Chen, Xin-Yi Tang, Ya-Ting Li

**Affiliations:** Pediatric Intensive Care Unit, Department of Pediatrics, Third Affiliated Hospital of Sun Yat-sen University, No. 600, Tianhe Road, Guangzhou, 510630 P.R. China

**Keywords:** Capillary leak syndrome, Sepsis, Continuous renal replacement therapy, Premature, Case report

## Abstract

**Background:**

Capillary leak syndrome (CLS) is a rare disease characterized by profound vascular leakage and presents as a classic triad of hypotension, hypoalbuminemia and hemoconcentration. Severe CLS is mostly induced by sepsis and generally life-threatening in newborns, especially in premature infants. Continuous renal replacement therapy (CRRT) plays an important role of supportive treatment for severe CLS. Unfortunately, CRRT in preterm infants has rarely been well defined.

**Case presentation:**

We report the case of a 11-day-old girl with CLS caused by sepsis, who was delivered by spontaneous vaginal delivery (SVD) at gestational age of 25 weeks and 4 days, and a birth weight of 0.89 Kilograms(kg). The infant received powerful management consisting of united antibiotics, mechanical ventilation, intravenous albumin and hydroxyethyl starch infusion, vasoactive agents, small doses of glucocorticoids and other supportive treatments. However, the condition rapidly worsened with systemic edema, hypotension, pulmonary exudation, hypoxemia and anuria in about 40 h. Finally, we made great efforts to perform CRRT for her. Fortunately, the condition improved after 82 h’ CRRT, and the newborn was rescued and gradually recovered.

**Conclusion:**

CRRT is an effective rescue therapeutic option for severe CLS and can be successfully applied even in extremely-low-birth-weight premature.

**Supplementary Information:**

The online version contains supplementary material available at 10.1186/s13052-021-01067-8.

## Introduction

Capillary leak syndrome (CLS) is a rare disease characterized by increasing permeability of capillaries to proteins, which leads to the loss of protein-rich fluid from the intravascular to the interstitial spaces. CLS is a devastating condition associated with various diseases, and the most common cause in newborn is sepsis induced by severe infection [1]. The real incidence of CLS is unclear, because many cases of CLS may not be recognized or diagnosed. Despite the considerable progress in intensive care support, there is still up to 57% of mortality rate of sepsis related CLS in children [2]. Implementation of appropriate antimicrobial therapy is the basic treatment of CLS caused by sepsis. Whereas, effective fluid managements, including volume resuscitation and vasoactive drugs for stabilizing blood pressure, fluid-restrictive strategy to prevent fluid overload, and diuretic therapy to remove edema, are essential to improve the success rate of salvage. CRRT is required when marked anasarca or acute kidney injury (AKI) with oliguria develops [1]. Unfortunately, CRRT in preterm infants has rarely been well defined. In this report, we presented the case of an extremely-low-birth-weight premature who developed severe CLS due to sepsis at 11 days of age. She poorly responded to the conventional treatment, but finally survived with the application of CRRT. To our knowledge, she was the lightest patient to be treated with CRRT support as per the available reports. Therefore, CRRT is an effective rescue therapeutic for severe CLS, and can be successfully applied even in extremely-low-birth-weight premature.

## Case presentation

We report here a test-tube newborn girl, who was delivered by SVD at gestational age of 25 weeks and 4 days with a birth weight of 0.89 kg. The Apgar scores were 10 at 1, 5 and 10 min of life. Her mother underwent pregnancy with cervical incompetence and premature rupture of membranes (4 days before delivery) and was managed with antibiotic Cefoperazone-Sulbactam to prevent infection. The baby was admitted to neonatal intensive care unit (NICU) and soon it developed respiratory distress along with cyanosis. After a series of timely treatment including mechanical ventilation, pulmonary surfactant replacement, combination of Meropenem and Ampicillin and fluid replacement, the baby’s condition became better and stable.

However, on 11th day of life, the infant showed a sharp decline in her general condition. She was noted to have progressive anasarca, oliguria, phases of severe apnea, pulmonary hemorrhage, developed with tachycardia (heart rate, HR >170/min), hypoxemia (saturation of peripheral oxygen, SpO_2_ 60–85%) and hypotension (blood pressure, BP 36/22 (28) mmHg, sometimes was too low to be measured). Blood gas analysis showed pH 7.15, arterial partial pressure of oxygen (PaO_2_) 60.4 mmHg, arterial partial pressure of carbon dioxide (PaCO_2_) 55.5 mmHg, blood lactate (Lac) 0.4 mmol/L, base excess (BE) -8.9 mmol/L. Laboratory investigation suggested impaired renal function (serum creatinine (Cr) 46umol/L, blood urea nitrogen (BUN) 22.52 mmol/L, estimated glomerular filtration rate (eGFR) 51.02 ml/min/1.73 m^2^). C-reactive protein (CRP) was 0.4 mg/L. X-ray revealed bilateral infiltrates in both lung fields. The diagnoses of “pneumonia, septic shock and CLS” were considered. Rapid infusion of normal saline, albumin and hydroxyethyl starch were administrated immediately, following with the treatment of a series of vasoactive agents (Milrinone, cedilanid, dopamine and noradrenalin), adjusted antibiotics to Meropenem and Piperacillin-tazobactam, mechanical ventilation and other supportive treatments (small doses of hydrocortisone (6 mg iv drip q6h × 2 times), low-dose heparin (0.5 mg/kg·d × 1d), Ulinastatin, blood products for infusion (red blood cell, plasma and cryoprecipitate)). Nevertheless, she responded so poorly that sufficient fluid resuscitation was hard to maintain hemodynamic stability. In addition, systematic edema, oliguria and pulmonary hemorrhage were deteriorated, following with continuous fever and diarrhea with a little blood per rectum on next day. Laboratory reevaluation showed pH 7.311, PaO2 66.9 mmHg, PaCO2 36.6 mmHg, Lac 3.1 mmol/L, BE − 7.1 mmol/L, Cr 173umol/L, BUN 22.96 mmol/L, eGFR 41.56 ml/min/1.73m^2^, CRP 2.0 mg/L, Procalcitonin (PCT) 32.19 ng/ml. Since the conventional treatments have been exhausted for the baby, we should spare no effort to control CLS. The patient was shifted to CRRT at 12th day of age, and her parents place great hopes on CRRT. Blood circulation access used umbilical vein catheter (UVC, 4Fr Catheters Ombilicaux, PRODIMED, France) to play the role of hemofiltration channel. In addition, red blood cells transfusion was induced before establishment of CRRT support. The prescription for CRRT was summarized in Table 1.

**Table 1:** Prescription for CRRT

Parameter	Value
Device brand	Asahikasei Plasauto
Hemofilter	Polysulfone hemofilter with surface area of 0.3 m^2^
Standard tubing set	Total extracorporeal volume (hemofilter and tubing) 80 ml
Precharge	Plasma precharge of extracorporeal circuit
Blood flow rate	3 ml/min
Modality	Continuous venovenous hemofiltration (CVVH)
Replacement fluid flow rate	35 ml/h
Anticoagulation	Citrate
Ultrafiltration plan	Even fluid balance to start, 0-10 ml/h

**Fig. 1:**
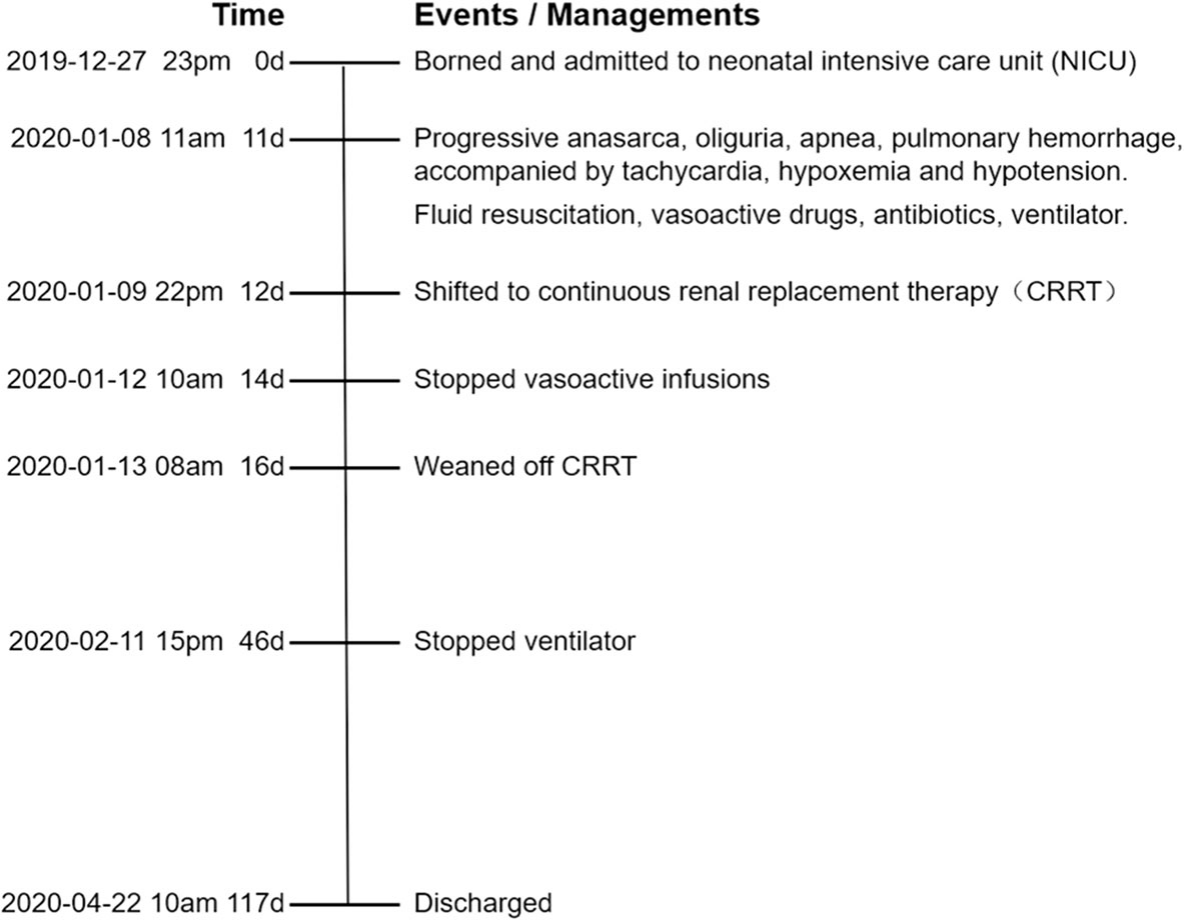
Timeline of the events and management details of CLS secondary to sepsis in our case

BP of the baby showed a mild drop at initiation stage of the CRRT circuit, but soon got stable with an increasing dose of vasoactive infusions. In the course of the initial 8 h, the baby tolerated ultrafiltration of all infused volumes, maintaining a “net zero” fluid balance, the vital signs improved while the vasoactive infusions could be reduced gradually; then we increased the ultrafiltration rate to 5–10 ml/h and achieved a net fluid loss of about 200 ml during 82 h’ CRRT. She was weaned off CRRT due to the blockage of UVC. Fortunately, the condition of the baby turned much better with acceptable hemodynamic conditions. Meanwhile, the urine volume gradually increased to 4-6 ml/h with the use of furosemide, and the edema continued to reduce. Thereafter we continued to carry out comprehensive treatments for the preterm, finally we discharged her with completely recovery after 117 days’ hospitalization (weight gain from 0.89 kg to 2.89 kg).(Timeline of the case is shown in Fig. 1).

## Discussion

CLS is a complicated process induced by various diseases. Cumulative studies have shown the probable mechanism of CLS sharing the similar underlying pathophysiologic abnormality, including hyper cytokinemia which leads to capillary leak, persistent excessive inflammatory reaction triggers oxygen metabolism disorder and multiple organ dysfunction syndrome (MODS) [3–5]. It often requires strenuous fluid resuscitation to maintain a basic blood perfusion, which is also a double-edged sword that brings to fluid overload.

As an effective treatment in many critical diseases, CRRT can not only achieve continuous ultrafiltration and solute clearance, but also be useful for the removal of inflammatory mediators, including proinflammatory cytokines, chemokines, and complements [6, 7]. CRRT plays an important role of supportive treatment for severe CLS [8]. Herein, we reported a 11-day-old premature who suffered from severe CLS induced by sepsis and soon developed to MODS. We performed CRRT and fortunately, the baby gradually recovered after 82 h’ CRRT. To our knowledge, she was the lightest patient to be treated with CRRT support as per the available reports. Though CRRT has been widely used in adults and older children, it remains a challenge in newborn, especially for extremely-low-birth-weight premature [5, 9].When it comes to the success rate of performing CRRT, we acknowledged that there are two aspects: one contains the device, such as a designed mode for this population and convenient sized central venous access to accommodate adequate blood flow; the other involves appropriate anticoagulation regimens [10]. In addition, the total blood volume of the newborn is relatively small, which make it difficult to keep hemodynamic stability during CRRT. According to our experience, red blood cell transfusion before starting CRRT to keep a good HGB and sufficient circulation perfusion and increasing the dosage of vasoactive drugs to keep a good BP are both important to carry out CRRT successfully.

The optimal timing of CRRT application in premature has not been established yet, so it’s still difficult to decide when to initiate CRRT support in these critically ill infants. Siddall, E et al. [1] suggested that anasarca and AKI with oliguria were indications for CRRT support. In the study of Noh ES et al. [11], the most common cause for CRRT in preterm infants were sepsis, necrotizing enterocolitis and inborn error of metabolism. Only 15% (5/33) of the preterm who received CRRT in the NICU survived. They found that there were significant differences between survivors and non-survivors in fluid overload > 10% (82% vs. 20%, *p* = 0.013) and inotropic support (5% vs. 28%, *p* = 0.011) at CRRT initiation stage, but not in BUN and creatinine. Another research about neonatal hyperammonemia suggested that the pre-CVVH physiological condition of the neonates in this cohort was the main determinant of outcome [12]. So, if there are suitable equipment and well-experienced medical staff, it may be better to start CRRT as early as possible when there is a tendency of irreversible fluid overload or other extremely internal environment disorder, rather than waiting for the worst condition and apply CRRT as a remedial measure.

## Conclusion

CRRT is an effective rescue therapeutic for severe CLS due to sepsis, and can be successfully applied even in extremely-low-birth-weight premature. To improve the success rate of salvage, prompt diagnosis, effective fluid management, and backup with CRRT support by an experienced intensive care team are pivotal to survival for premature neonates with severe CLS.

## Supplementary Information

**Additional file 1: Table 1S.** Reference Ranges of Laboratory Parameters.

## Data Availability

Data sharing is not applicable to this article as no datasets were generated or analysed during the current study.

## Abbreviations

CLS: Capillary leak syndrome
Kg: Kilograms
CRRT: Continuous renal replacement therapy
SVD: Spontaneous vaginal delivery
AKI: Acute kidney injury
NICU: Neonatal intensive care unit
UVC: Umbilical vein catheter
HR: Heart rate
SpO_2_: Saturation of peripheral oxygen
BP: Blood pressure
PaO_2_: Arterial partial pressure of oxygen
PaO_2_: Arterial partial pressure of carbon dioxide
Lac: Lactate
BE: Base excess (BE) -8.9 mmol/L.
Cr: Creatinine
BUN: Blood urea nitrogen
eGFR: Estimated glomerular filtration rate
CRP: C-reactive protein
CVVH: Continuous venovenous hemofiltration
MODS: Multiple organ dysfunction syndrome
