# Supplementary material for: Continuous renal replacement therapy rescued life-threatening capillary leak syndrome in an extremely-low-birth-weight premature: a case report
Source: Ital J Pediatr. 2021 May 26;47:116. doi: 10.1186/s13052-021-01067-8 (PMC8157435; doi:10.1186/s13052-021-01067-8)
Supplement: Supplementary file 1 — Additional file 1: Table 1S. Reference Ranges of Laboratory Parameters. [file 13052_2021_1067_MOESM1_ESM.docx]

Table 1S Reference Ranges of Laboratory Parameters

| Laboratory Parameters | Reference Ranges |
| --- | --- |
| pH | 7.35-7.45 |
| arterial partial pressure of oxygen (PaO2) | 95-105mmHg |
| arterial partial pressure of carbon dioxide (PaCO2) | 35-45mmHg |
| blood lactate (Lac) | 0.5-1.6mmol/L |
| base excess (BE) | ±2.3mmol/L |
| renal function (serum creatinine (CREA) | 31.8-91.0umol/L |
| blood urea nitrogen (BUN) 22.52mmol/L | 2.4-8.2mmol/L |
| estimated glomerular filtration rate (eGFR) | / |
| C-reactive protein (CRP) | 0-6.0mg/L |
| Procalcitonin (PCT) | 0-0.05ng/ml |
